# Factors related to community pharmacists’ intention to provide collaborative deprescribing service for fall-risk medications in older adults: a factorial and theory-based cross-sectional study

**DOI:** 10.1007/s11096-026-02173-5

**Published:** 2026-06-23

**Authors:** Zeynep Sayın, Ilgın Asena Cebeci, Mesut Sancar, Betul Okuyan

**Affiliations:** 1 Clinical Pharmacy Department, Institute of Health Science, Faculty of Pharmacy, Marmara University, Istanbul, Türkiye; 2 Department of Biostatistics, Institute of Health Science, Hacettepe University, Ankara, Türkiye; 3 School of Medicine, Department of Biostatistics, Hacettepe University, Ankara, Türkiye; 4 Department of Clinical Pharmacy, Faculty of Pharmacy, Marmara University, Istanbul, Türkiye

**Keywords:** Deprescribing, Factorial Survey, Falls, Older Adults, Pharmaceutical Care, Potentially Inappropriate Medications, Pharmacist, Theory of Planned Behaviour

## Abstract

**Introduction:**

Collaborative deprescribing of fall-risk-increasing medication by community pharmacists and family physicians can reduce falls in older adults. No such exists in Türkiye.

**Aim:**

To identify factors related to community pharmacists’ intention (1) to provide collaborative deprescribing service for fall-risk medications in older adults, and (2) to discuss older adults’ potential fall-risk medications with the family physician.

**Method:**

In this cross-sectional study, a web-based survey was conducted between November 2023 and February 2024 among Turkish community pharmacists recruited using convenience sampling. The questionnaire included items at the respondent level (including a theory of planned behaviour-based questionnaire) and the vignette level (including factorial vignettes). The factors related to their intention to perform two behaviours were evaluated: (1) providing collaborative deprescribing service and (2) discussing older adults’ potential fall-risk medications with the family physician. Multilevel linear mixed-effects models were used.

**Results:**

Based on responses from 398 community pharmacists (response rate of 93.2%), the intention to provide collaborative deprescribing service was related to their knowledge test score, self-reported rate of older adults served in the community pharmacy (61–80% category), their scores of subjective norm, self-efficacy, and perceived behavioural control (*p* < 0.05). Community pharmacists’ intention to discuss older adults’ potential fall-risk medications with the family physician was related to being male, younger age, their experience on patient-centred services, their higher scores of subjective norm, self-efficacy, and perceived behavioural control, and lower scores of attitude (*p* < 0.05). At vignette level, both intentions were linked to having a history of falls and the number of patients waiting in the community pharmacy (*p* < 0.05).

**Conclusion:**

A collaborative deprescribing service for fall-risk-increasing medications in older adults could be implemented by addressing key determinants of community pharmacists’ intention at both respondent and vignette levels, including subjective norm, self-efficacy, perceived behavioural control, attitude, workload-related factors, and patient clinical history.

**Supplementary Information:**

The online version contains supplementary material available at https://doi.org/10.1007/s11096-026-02173-5.

## Impact statements

- Factors related to community pharmacists’ intention to provide collaborative deprescribing service for fall-risk medications in older adults and discuss older adults’ potential fall-risk medications with the family physician could be identified by multilevel linear mixed-effects models including items at the respondent level and the vignette level.
- Community pharmacists’ intention to provide collaborative deprescribing service for fall-risk medications in older adults and discuss older adults’ potential fall-risk medications with the family physician were related to subjective norm, self-efficacy, perceived behavioural control, attitude, workload-related factors, and patient clinical history.

## Introduction

Falls are a serious and prevalent health issue among older adults, affecting 26.5% globally and 28.5% in Türkiye [1, 2]. Medications, particularly fall-risk-increasing medications (FRIDs) like sedatives and antidepressants, are major modifiable risk factors for falls in older adults [3–7]. Notably, using multiple medications or ≥ 2 FRIDs independently increases the risk of fall [3]. Persistently high FRIDs use (65–93%) among older adults, even post-fall [8], underscores the need for systematic deprescribing as a core component of guideline-based falls prevention [3, 9]. The decision-making process in fall prevention is complex and requires interprofessional collaboration [10]. However, no collaborative deprescribing service currently exists between community pharmacists and family physicians in Türkiye. Community pharmacists play a central role in providing pharmaceutical care services related to FRIDs, including screening and deprescribing [11]. Research indicates that pharmacist-led deprescribing interventions in collaborative primary care settings are highly acceptable to both patients and healthcare professionals while effectively reducing the use of high-risk medications [12]. Pharmacist-led interventions and team-based collaborations are proven to reduce FRIDs and improve the identification of high-risk older adults [13–16].

Regardless of funding or policy endorsement, the transition of services into practice is primarily driven by pharmacists’ intention and their perceived capability to implement them [17, 18]. The intention of community pharmacists to provide patient-centred services, such as fall prevention services, is affected by many different factors, such as insufficient training, lack of time, limited access to patient records, lack of reimbursement, limited interprofessional collaboration, and low patient demand or awareness [19–21]. Literature consistently identifies time constraints, limited multidisciplinary collaboration, and lack of reimbursement as the primary barriers to fall prevention services [22–24]. Identifying the most significant of these obstacles is crucial for designing interventions that enhance pharmaceutical care for older adults [19–21]. The Theory of Planned Behavior (TPB) is a well-established psychological framework widely utilized to identify and predict healthcare professionals’ intentions and clinical practices, particularly within community pharmacy settings. TPB constructs (attitudes, subjective norms, and perceived behavioural control) are associated with behavioural intention [19, 21, 25, 26]. The behavioural determinants of community pharmacists’ intention to provide collaborative deprescribing for FRIDs in older adults have not been previously studied using both using theory-based and factorial surveys. TPB-based factorial surveys effectively evaluate pharmacists' intentions under realistic scenarios by integrating randomized clinical vignettes [27, 28]. This methodology enables a dual analysis of situational factors and individual behavioural constructs, providing a robust framework for investigating intentions toward collaborative deprescribing services [25, 29–31].

## Aim

The study aimed to identify factors related to community pharmacists’ intention to provide collaborative deprescribing service for fall-risk medications in older adults and their intention to discuss these medications with family physicians by using a factorial survey and a TPB model.

## Method

### Study design, setting, participants

This cross-sectional web-based survey was conducted between November 2023 and February 2024. Participants included actively practicing licensed community pharmacists in Türkiye recruited through convenience sampling.

### Data collection

The survey was web-based but administered in person, with participants completing it on a tablet using an online platform during face-to-face recruitment. The questionnaire consisted of four sections: (1) demographic and professional practice questions, (2) a knowledge test regarding PIMs and FRIDs, (3) the TPB-based questionnaire for providing the pharmaceutical care service, including steps of a collaborative deprescribing service for fall-risk medications in older adults, and (4) five factorial vignettes and one standard vignette. The total estimated time to complete the full survey was approximately 15–20 min. The content validity of the items (including factorial vignettes) was established through a review by two clinical pharmacists and two public health specialists. The pilot test was conducted among five community pharmacists.

#### Demographic and professional practice questions

Information on the demographic and professional characteristics of community pharmacists was collected, including age, sex, self-reported rate of older adults served in the community pharmacy, and experience in patient-centred services, assessed by participation in the pharmaceutical care project led by the Turkish Pharmacists’ Association (TPA) for older adults [32].

#### Knowledge test

The knowledge test was developed by the research team and included three questions centred on PIMs and FRIDs in older adults. The instrument was developed based on existing literature [33–35]. Each item had three options: true, false, and “I don’t know”. Knowledge test scores were calculated based on the total number of correct answers, with higher scores indicating higher knowledge levels.

#### TPB-based questionnaire

A TPB-based questionnaire was translated and culturally adapted based on TPB literature and a guidebook for health services researchers conducting TPB studies [36–39]. The developers were permitted to adapt the Turkish version of this questionnaire [39]. The TPB-based questionnaire consisted of 26 items assessing intention (4 items), attitude (8 items), subjective norm (3 items), self-efficacy (3 items), and perceived behavioural control (8 items). Turkish translation and cultural adaptation (including forward and back translation) were performed following the guidelines of the International Society for Pharmacoeconomics and Outcomes Research (ISPOR) Task Force [40]. A pilot study was conducted with 20 community pharmacists to ensure clarity. Information about the pharmaceutical care service (definitions and steps of the collaborative deprescribing service for fall-risk medications) was provided at the beginning of the questionnaire. A 5-point Likert scale ranging from strongly disagree (1) to strongly agree (5) was used. For the test–retest reliability, thirty community pharmacists completed the questionnaire at baseline and again after two weeks.

#### Development of vignette skeleton and variables for factorial survey

Patient-related (age, sex, history of falls), medication (total number of medications used, duration of medication utilization, and type of medication), and environmental variables (the number of patients waiting in the community pharmacy) were selected from the literature [25, 27, 35, 41]. Specific medications (paroxetine, quetiapine, metoprolol, and valproic acid) were selected based on clinical relevance, frequency in primary care, and expert consensus [42–45].

Vignettes were embedded with randomly varying factors (Table 1), generated via Microsoft Excel to create 11,520 unique combinations. The survey programming randomly assigned each respondent five factorial vignettes drawn from a pool of 11,520 possible scenarios, ensuring that each respondent received a unique set of vignettes. Additionally, all respondents were presented with one standard vignette. Like previous factorial survey studies, each survey included five factorial vignettes and one standardized vignette to reduce response fatigue and the potential development of response heuristics [27, 28, 41, 46]. For each vignette, the pharmacist was instructed to rank the intention of their behaviours on a scale of 0–10 (0- not at all likely, 10-extremely likely) for each of two questions: (1) providing collaborative deprescribing service for fall-risk medications in older adults, and (2) discussing this older adult’s potential fall-risk medications with the family physician. These two questions were generated based on published pharmaceutical care services to prevent fall risk in older adults [35]. For the standard vignette, one more question was ranked to evaluate the realism of the scenario in relation to their practice (possible range 0–10). The vignette design was constructed as a fully orthogonal factorial design, where all attribute levels were independently and randomly combined by the survey algorithm to ensure that vignette dimensions were not systematically correlated. The orthogonality of the vignette design was assessed by examining the pairwise correlations between dimensions across unique profiles. Categorical factors were dummy-coded, and the correlation matrix was computed on the design matrix.

**Table 1:** Characteristics of vignette factors

Vignette factor	Values
*Patient-related factors*	
Age	1–68 years old
	2–76 years old
	3–87 years old
Sex	1- Female
	2- Male
History of falls	1- Yes
	2- No
*Medication-related factors*	
Total number of medications used	2 3 4 5 6 7 8 9
Type of medication	1- Paroxetine 2- Quetiapine 3- Metoprolol 4- Valproic acid
Duration of medication utilization	1- 2 months 2- 6 months 3- 1 year 4- 2 years 5- 5 years
*Environmental factors*	
Number of patients waiting in the community pharmacy	0 1 2 3 4 5
**Vignette Skeleton**	
You are dispensing the prescription of < < insert value for age > > < < insert value for sex > > patient There is < < insert value for type of medication > > in the prescription Duration of medication utilization = < < insert value for duration of medication utilization [month/year] > > History of falls within the last year = < < insert value for history of falls > > Total number of medications used = < < insert value for total number of medications used > > Number of patients waiting in the community pharmacy = < < insert value for number of patients waiting in the community pharmacy > >	
**Standard vignette**	
You are dispensing the prescription of < < 76 years old > > < < female > > patient There is < < paroxetine > > in the prescription Duration of medication utilization = < < 2 years > > History of falls within the last year = < < yes > > Total number of medications used = < < nine > > Number of patients waiting in the community pharmacy = < < three > >	

*The vignette universe is created by 11,520 possible combinations of values for each vignette factor (3 × 2 × 2 × 8 × 4 × 5 × 6) embedded within the vignette skeleton

### Sample size calculation

Instead of prior sample size calculation [47, 48], the sensitive power analysis was done for multilevel linear mixed-effects models. The sensitivity power analysis was conducted using G*Power 3.1.9.7 to determine the minimum detectable effect size for a fixed-model linear multiple regression (R^2^ deviation from zero).

### Statistical analysis

Descriptive statistics—frequencies, percentages, and medians [IQR]—were analysed using SPSS 29.0 and jamovi (v2.4.14). Data normality was assessed via the Kolmogorov–Smirnov test. Psychometric evaluations of the knowledge test and TPB-based questionnaire are provided in the Supplementary Material. Multilevel linear mixed-effects models were performed in R (v4.4.2) using Restricted Maximum Likelihood (REML) to identify predictors of behavioural intention (Model 1 & 2). Variables were modelled at two levels: vignette (Level 1) and respondent (Level 2). Continuous variables were z-standardized before analysis. Model fit was evaluated using Akaike Information Criterion (AIC), AICc (corrected AIC), Bayesian Information Criterion (BIC), conditional/marginal R^2^, root mean square error (RMSE), and sigma. Intraclass Correlation Coefficients (ICC) assessed respondent-level variability, with ICC > 0.500 indicating that a substantial portion of the total variance was attributable to differences between respondents rather than the vignettes themselves. Multicollinearity was assessed using Variance Inflation Factors (VIF) and tolerance values; thresholds of VIF < 5 and tolerance > 0.50 were applied to ensure acceptable collinearity. Statistical significance was set at *p* < 0.05, with interpretation based on effect direction, magnitude, and 95% confidence intervals (CIs).

### Ethics approval

The study protocol was approved by the Marmara University Institute of Health Ethics Committee (approval date and number: 18.10.2021/120). The participants completed an electronic informed consent form. TPA permitted this study to be conducted among Turkish community pharmacists.

## Results

A total of 427 community pharmacists were invited to participate in the survey; 29 did not provide consent, and 398 completed the study, yielding a response rate of 93.2%. There was no missing data. Respondent characteristics are summarized in (Table 2). Detailed psychometric properties for the knowledge test and the TPB-based questionnaire are provided in the Supplementary Material. In the sensitivity power analysis, the minimum detectable effect size at α = 0.05 and 0.80 power was calculated as f^2^ = 0.059 with a total sample of N = 398 and 24 predictors. This indicated that the study had the sensitivity to detect effects slightly above the small level. In testing level of orthogonality, the maximum absolute correlation was |r|= 0.335, and the mean absolute correlation was 0.052.

**Table 2:** Characteristic of community pharmacists (n = 398)

Age (years) median [IQR]	37 [30.0–46.0]
Sex n (%)	
Female	220 (55.3)
Male	178 (44.7)
Self-reported rate of older adults served in the community pharmacy n (%)	
< 20	70 (17.6)
21–40	116 (29.1)
41–60	129 (32.4)
61–80	81 (20.4)
> 80	2 (0.5)
Taking part in the pharmaceutical care project led by TPA* n (%)	
Yes	58 (14.6)
No	340 (85.4)

TPA: Turkish Pharmacists’ Association * to assess their experience on patient-centred services

### Findings of multilevel linear mixed-effects models

Table 3 presents multilevel linear mixed-effects models testing the associations of vignette level variables (level 1) and respondent level variables (level 2) on the intention to both provide collaborative deprescribing service for fall-risk medications in older adults and their intention to discuss these medications with family physicians in response to factorial vignettes. All community pharmacists (n = 398) completed questions about all five randomly assigned vignettes and one standard vignette. A total of 1990 vignettes were included in the analyses. Respondents provided a median rating of 6.00 [4.00–7.00] for the realism of the scenario (possible range 0–10) in relation to their practice in the standard vignette. The VIF values for all variables of the two models ranged from 1.0 to 1.4.

**Table 3:** Multilevel linear mixed-effects models testing the associations of vignette level variables (level 1) and respondent level variables (level 2) on the intention to perform collaborative deprescribing service for fall-risk medications in older adults in response to factorial vignettes

	Model 1- Intention to provide collaborative deprescribing service for fall-risk medications in older adults						Model 2- Intention to discuss older adults’ potential fall-risk medications with the family physician ^b^					
	β	b	SE	t	[95% CI]	*p*-value	β	b	SE	t	[95% CI]	*p*-value
**Intercept**		5.131	0.255	20.150	4.631–5.631	< 0.001		4.618	0.288	16.013	4.052–5.185	< 0.001
***Level 1- Vignette factors***												
Age	0.040	0.040	0.037	1.066	− 0.033–0.113	0.287	0.007	0.014	0.035	0.417	− 0.054–0.083	0.676
Female	0.082	0.122	0.074	1.646	− 0.023–0.268	0.100	0.020	0.061	0.069	0.878	− 0.075–0.196	0.380
History of falls	0.494	0.733	0.075	9.776	0.586–0.880	< 0.001	0.188	0.557	0.070	7.975	0.420–0.694	< 0.001
Total number of medications used	0.001	0.001	0.037	0.021	− 0.073–0.074	0.984	− 0.009	− 0.020	0.035	− 0.568	− 0.088–0.049	0.570
*Type of medication*												
*Quetiapine*	0.044	0.065	0.105	0.612	− 0.142–0.271	0.541	0.044	0.129	0.098	1.318	− 0.063–0.322	0.188
*Metoprolol*	− 0.106	− 0.158	0.105	− 1.507	− 0.363–0.048	0.132	− 0.013	− 0.039	0.098	− 0.396	− 0.230–0.153	0.692
*Valproic Acid*	− 0.014	− 0.021	0.106	− 0.203	− 0.228–0.186	0.840	0.063	0.188	0.098	1.907	− 0.005–0.381	0.057
*Duration of medication utilization*												
*6 months*	− 0.115	− 0.170	0.119	− 1.431	− 0.403–0.063	0.153	0.035	0.104	0.111	0.942	− 0.113–0.321	0.346
*1*-*year*	0.055	0.081	0.117	0.695	− 0.148–0.311	0.487	0.034	0.102	0.109	0.935	− 0.112–0.316	0.350
*2*-*years*	− 0.081	− 0.120	0.118	− 1.013	− 0.352–0.112	0.311	− 0.002	− 0.006	0.110	− 0.053	− 0.222–0.211	0.958
*5*-*years*	− 0.020	− 0.030	0.118	− 0.256	− 0.261–0.201	0.798	0.018	0.053	0.110	0.481	− 0.162–0.268	0.630
Number of patients waiting in the community pharmacy	− 0.205	− 0.348	0.037	− 9.288	− 0.421– − 0.274	< 0.001	− 0.070	− 0.239	0.035	− 6.838	− 0.307– − 0.170	< 0.001
***Level 2- Respondent factors***												
Age (years)	− 0.077	− 0.011	0.009	− 1.203	− 0.029–0.007	0.230	− 0.097	− 0.028	0.011	− 2.579	− 0.049– − 0.007	0.010
Female	− 0.206	− 0.306	0.178	− 1.723	− 0.655–0.043	0.086	− 0.151	− 0.448	0.209	− 2.143	− 0.858– − 0.037	0.033
*Self*-*reported rate of older adults served in the community pharmacy (%)*												
*21–40*	− 0.073	− 0.108	0.263	− 0.411	− 0.624–0.408	0.682	− 0.040	− 0.120	0.309	− 0.388	− 0.726–0.487	0.698
*41–60*	0.133	0.198	0.262	0.756	− 0.317–0.712	0.450	0.003	0.008	0.308	0.026	− 0.597–0.613	0.979
*61–80*	0.535	0.794	0.291	2.726	0.221–1.366	0.007	0.183	0.541	0.342	1.581	− 0.132–1.214	0.115
> *80*	1.599	2.370	1.249	1.898	− 0.085–4.826	0.058	0.705	2.090	1.468	1.423	− 0.797–4.976	0.155
Taking part in the pharmaceutical care project led by TPA (%)*	0.141	0.210	0.255	0.821	− 0.292–0.711	0.412	0.274	0.811	0.300	2.704	0.221–1.401	0.007
Knowledge test score	0.255	0.255	0.090	2.826	0.078–0.433	0.005	–0.062	− 0.124	0.106	− 1.173	− 0.333–0.084	0.242
Attitude score	− 0.063	− 0.254	0.168	− 1.509	− 0.584–0.077	0.132	− 0.105	− 0.844	0.197	− 4.272	− 1.232– − 0.455	< 0.001
Subjective norm score	0.271	0.407	0.126	3.221	0.159–0.656	0.001	0.275	0.825	0.149	5.550	0.533–1.117	< 0.001
Self − efficacy score	0.139	0.417	0.126	3.316	0.170–0.664	< 0.001	0.098	0.588	0.148	3.982	0.298–0.878	< 0.001
Perceived behaviour control score	0.301	0.481	0.122	3.943	0.241–0.721	< 0.001	0.229	0.733	0.143	5.108	0.451–1.015	< 0.001

β=standardized coefficients. b=unstandardized coefficients; CI: confidence interval

Values in bold were statistically significant at *p* <0.05

In Model 1: conditional R^2^ = 0.616, marginal R^2^ = 0.196, RMSE = 1.377, sigma = 1.511, AIC = 8154.7, AICc=8155.5 and BIC = 8305.8

In Model 2: conditional R^2^ = 0.733, marginal R^2^ = 0.228, RMSE = 1.265, sigma = 1.398, AIC = 8033.9, AICc= 8034.7 and BIC = 8185.0

*To assess their experience on patient-centred services

### Community pharmacists’ intention to provide collaborative deprescribing service for fall-risk medications in older adults

The null model's ICC of 0.523 indicates that 52.3% of the response variance stems from respondent-level differences. At vignette level, history of falls (β = 0.49; b = 0.73, SE = 0.08, t = 9.78, 95% CI [0.59, 0.88], *p* < 0.001) strongly increased community pharmacists’ intention to provide this collaborative deprescribing service, indicating that, on average, scenarios involving patients with a prior fall were associated with a 0.73-point higher intention score. In contrast, the number of patients waiting in the community pharmacy (β =  − 0.21; b =  − 0.35, SE = 0.04, t =  − 9.29, 95% CI [− 0.42, − 0.27], *p* < 0.001) significantly reduced community pharmacists’ intention to provide this collaborative deprescribing service.

At the respondent level, knowledge test score (β = 0.26; b = 0.26, SE = 0.09, t = 2.83, 95% CI [0.08, 0.43], *p* = 0.005) and self-reported rate of older adults served in the community pharmacy between 61–80% range (β = 0.54; b = 0.79, SE = 0.29, t = 2.73, 95% CI [0.22, 1.37], *p* = 0.007) significantly increased the intention to provide this collaborative deprescribing service.

Psychometric determinants demonstrated significant positive effects for subjective norm (β = 0.27; b = 0.41, SE = 0.13, t = 3.22, 95% CI [0.16, 0.66], *p* = 0.001), self-efficacy (β = 0.14; b = 0.42, SE = 0.13, t = 3.32, 95% CI [0.17, 0.66], *p* < 0.001), and perceived behavioural control (β = 0.30; b = 0.48, SE = 0.12, t = 3.94, 95% CI [0.24, 0.72], *p* < 0.001).

### Community pharmacists’ intention to discuss older adults’ potential fall-risk medications with the family physician

The null multilevel model's ICC of 0.654 indicates that 65.4% of the response variance is due to respondent-level differences. At vignette level, the most notable effect was observed for the history of falls (β = 0.19; b = 0.56, SE = 0.07, t = 7.98, %95 CI [0.42, 0.69], *p* < 0.001), indicating that community pharmacists’ intention to discuss older adults’ potential fall-risk medications with family physicians by approximately 0.56 points in scenarios involving patients with a prior fall. The number of patients waiting in the community pharmacy exhibited a significant negative effect (β =  − 0.07; b =  − 0.24, SE = 0.04, t =  − 6.84, %95 CI [− 0.31, − 0.17], *p* < 0.001).

At the respondent level, sex (β =  − 0.15; b =  − 0.45, SE = 0.21, t =  − 2.14, %95 CI [− 0.86, − 0.04], *p* = 0.033) exerted a significant negative effect, with female pharmacists exhibiting, on average, 0.45 points lower intention to discuss with the family physician than their male colleagues. Age also demonstrated a significant negative association (β =  − 0.10; b =  − 0.03, SE = 0.01, t =  − 2.58, %95 CI [− 0.05, − 0.01], *p* = 0.010). Taking part in the pharmaceutical care project led by TPA had a significant positive effect (β = 0.27; b = 0.81, SE = 0.30, t = 2.70, %95 CI [0.22, 1.40], *p* = 0.007). Subjective norm (β = 0.28; b = 0.83, SE = 0.15, t = 5.55, %95 CI [0.53, 1.12], *p* < 0.001), self-efficacy (β = 0.10; b = 0.59, SE = 0.15, t = 3.98, %95 CI [0.30, 0.88] *p* < 0.001), and perceived behavioural control (β = 0.23; b = 0.73, SE = 0.14, t = 5.11, %95 CI [0.45, 1.02] *p* < 0.001) showed strong positive effects and attitude exhibited a significant negative effect (β =  − 0.11; b =  − 0.84, SE = 0.20, t =  − 4.27, %95 CI [− 1.23, − 0.46], *p* < 0.001).

## Discussion

In this theory-based cross-sectional study, pharmacists’ intention to provide collaborative deprescribing for fall-risk medications was related to fall-risk knowledge, self-reported rate of older patients (61–80%), and TPB constructs except attitudes. Intention to discuss older adults’ potential fall-risk medications with physicians was associated with demographics of pharmacists, their patient-centred experience, and all TPB constructs. At the vignette level, both intentions were related to patients’ clinical history and pharmacy workload. At the vignette level, a history of falls was the primary positive determinant for both service provision and physician consultation, highlighting the impact of clinical risk awareness on intention. Since patients often underreport falls or are unaware of medication risks, targeted patient counselling during reviews is essential [11]. Consequently, international guidelines and systematic screening tools should identify at-risk patients and facilitate deprescribing discussions that might otherwise be overlooked [14, 49].

Increased workload and time pressure, specifically high patient volume and dispensing loads, significantly reduce pharmacists' intention to provide services, effectively obstructing comprehensive pharmaceutical care [22, 50]. The lack of significant effects for other vignette variables, such as type of medication, suggests insufficient differentiated knowledge of FRIDs among pharmacists or methodology limitations. This highlights the need for targeted educational interventions and refined vignette designs in future research.

Knowledge test scores and a higher rate of older adults served positively influenced the intention to provide collaborative deprescribing services. This aligns with research linking higher knowledge [51, 52] and greater prescription volume or patient contact [17] with increased willingness to deliver extended pharmaceutical care.

Younger and male community pharmacists were more likely to discuss fall-risk medications with physicians. While gender-based professional risk-taking [53] may contribute, factors like workload or practice patterns should also be considered. Furthermore, higher collaboration among younger pharmacists aligns with their documented openness to interprofessional care [54]. Consistent with previous research [55], TPA projects and training improved communication; however, the cross-sectional nature of the study may indicate correlation rather than causation.

Among psychometric determinants, perceived behavioural control, subjective norm, and self-efficacy demonstrated significant positive effects on the community pharmacists’ intention of providing this collaborative deprescribing service and discussing with the family physician. These findings align with previous studies [19, 56, 57]. Attitude unexpectedly showed a significant negative effect on pharmacists' intention to discuss deprescribing with family physicians, contrasting with prior studies where it positively influenced medical adherence monitoring [25, 27, 41]. These findings suggest that attitude alone is often insufficient to predict intention [19, 58–60]. In this study, the attitude-intention association was moderated by subjective norms and perceived behavioural control; specifically, when individuals feel unable to perform a behaviour, positive attitudes have a diminished impact on intention. Furthermore, social pressure and self-efficacy frequently demonstrate stronger associations with intention than attitude [58, 61, 62]. Lower factor loadings for certain attitude items may have also compromised construct validity, potentially contributing to this unexpected negative association.

### Strengths and limitations

This study’s strengths include its factorial survey methodology and TPB-based questionnaire. Systematic variable variation and random allocation ensured high internal validity, while the design increased vignette-level observations, enhancing the precision and power of estimated effects. The vignettes were found largely independent in the testing level of orthogonality. According to findings of sensitivity power analysis, the sample size was robust enough for the complex regression models. However, a formal power analysis accounting for the multilevel structure was not conducted. The limitations of the study also include potential nonresponse or self-selection bias and limited generalizability because of convenience sampling across Türkiye. The lack of data on practice-related variables, including location, professional experience, and consultation facilities, precluded their use as covariates in the analysis. Additionally, while factorial vignettes evaluate behavioural intentions through hypothetical scenarios, they do not assess actual behaviour, potentially reflecting an intention-behaviour gap rather than real-world practice. While realism ratings were generally acceptable, the low first quartile of these scores indicates that some participants found scenarios unrealistic, potentially limiting the external validity of the findings. The limited availability of structured collaborative pharmaceutical care services in Türkiye and variability in community pharmacist–physician collaboration may have influenced participants’ responses. Face-to-face administration may have introduced social desirability bias, leading pharmacists to report more favourable intentions than those practiced in real-world settings. However, factorial vignettes may mitigate this bias compared to real-world environments with higher accountability demands [63]. Additionally, the vignettes may not have captured all factors influencing behavioural intention [31], with system-level factors potentially being omitted during selection. The vignette development process was reviewed by two clinical pharmacists and two public health experts and a pilot test was conducted with five pharmacists, which may limit the content validity compared with studies that use broader pilot testing [27, 41]. The three-item knowledge assessment may have limited discriminatory power. Negative wording likely influenced response patterns, and the low internal consistency may affect measurement reliability, requiring cautious interpretation of the findings.

## Conclusion

This survey identified structural and knowledge-based barriers to collaborative deprescribing, most notably high workloads and difficulty distinguishing among medication types. Overcoming these barriers requires system-level changes, improved staffing, workflow optimization, and targeted professional development facilitated by decision-support tools. Notably, subjective norm consistently predicted the intention to collaborate, indicating that social and organizational expectations drive behaviour more effectively than individual attitudes alone. Therefore, interventions must prioritize strengthening pharmacist–physician collaboration and the broader interprofessional environment, especially since positive individual attitudes do not inherently translate into collaboration when perceived control or social support is lacking.

## Supplementary Information

Below is the link to the electronic supplementary material.

Supplementary file 1 (DOCX 2919 KB)

## Data Availability

The dataset is available from the corresponding author upon reasonable request.
