# Supplementary material for: Factors related to community pharmacists’ intention to provide collaborative deprescribing service for fall-risk medications in older adults: a factorial and theory-based cross-sectional study
Source: Int J Clin Pharm. 2026 Jun 23;48(5):1957–66. doi: 10.1007/s11096-026-02173-5 (PMC13569604; doi:10.1007/s11096-026-02173-5)
Supplement: Supplementary file 1 [file 11096_2026_2173_MOESM1_ESM.docx]

**Factors Related to Community Pharmacists’ Intention to Provide Collaborative Deprescribing Service for Fall-Risk Medications in Older Adults: a factorial and theory-based cross-sectional study**

Zeynep Sayın, Ilgın Asena Cebeci, Mesut Sancar, Betul Okuyan*

Zeynep Sayın ORCID ID: 0000-0002-8058-029X

Clinical Pharmacy Department, Institute of Health Science, Marmara University, Istanbul, Türkiye

Ilgın Asena Cebeci ORCID ID: 0000-0002-6982-2366

Department of Biostatistics, Institute of Health Science, Hacettepe University, Ankara, Türkiye

School of Medicine, Department of Biostatistics, Hacettepe University, Ankara, Türkiye

Mesut Sancar ORCID ID: 0000-0002-7445-3235

Department of Clinical Pharmacy, Faculty of Pharmacy, Marmara University, Istanbul, Türkiye

Betul Okuyan ORCID ID: 0000-0002-4023-2565

Department of Clinical Pharmacy, Faculty of Pharmacy, Marmara University, Istanbul, Türkiye

Corresponding author:

* Correspondence: Betul Okuyan (PhD), Associate Professor, ORCID ID: 0000-0002-4023-2565

Clinical Pharmacy Department, Faculty of Pharmacy, Marmara University, Istanbul, Türkiye.

e-mail address: betulokuyan@yahoo.com

**Supplement File**

***The psychometric analysis of knowledge test and TPB-based questionnaire***

Kuder-Richardson 20 was calculated to test the validity of the knowledge test. The factor analysis of the TPB-based questionnaire was assessed by using varimax rotation. The Kaiser–Meyer–Olkin (KMO) measure of sampling adequacy and Bartlett’s test of sphericity were also conducted. The Cronbach’s coefficient alpha was determined for each identified component based on factor analysis to show the internal consistency of the measures. For each component, the scores of the items were summed and divided by the total number of items within the component. The items with negative phrasing were reversed. Thus, each component was scored between 1 and 5. Test-retest reliability was determined by using the Wilcoxon test and the intraclass correlation coefficient (ICC [95% confidence interval, CI]). All zero-order correlations for all constructs of TPB-based questionnaire including the intention were found p<0.001.

**Supplement File Table 1.** Scores of knowledge test and its psychometric indices

| **Proportion of right response to the knowledge test n (%) *** | |
| --- | --- |
| Utilization of medications with high anticholinergic effects is appropriate in older adults with falls history. | 141 (35.4) |
| The rate of orthostatic hypotension in the older adults is not higher than adults who are not older. | 170 (42.7) |
| There is no need to identify risk of falls annually in older adults if their risk of fall has not been detected. | 171 (43.0) |
| **Total score of knowledge test Median [IQR]** | 1.00 [0.00-2.00] |

*Kuder Richardson 20=0.679

**Supplement File Table 2.** Factor loading of the TPB-based scale** (n=398)

|  | **Factor-1 Intention** | **Factor-2**  **Attitude** | **Factor-3**  **Subjective Norm** | **Factor-4**  **Self-Efficacy** | **Factor-5 Perceived Behaviour Control** |
| --- | --- | --- | --- | --- | --- |
| **Intention-1** I intend to provide this pharmaceutical care service* to older adults | 0.705 |  |  |  |  |
| **Intention-2** I intend to ask questions to older adults about their medicines and medical conditions during screening for the risk of falls | 0.625 |  |  |  |  |
| **Intention-3** I intend to specify drug-related problems that might enhance the risk of falls by conducting medication review (e.g., identifying medication use that increases the risk of falls) | 0.661 |  |  |  |  |
| **Intention-4** I intend to provide education and counseling service to older adults to reduce the risk of falls | 0.586 |  |  |  |  |
| **Attitude-1** Providing this pharmaceutical care service* enhances my professional development |  | 0.679 |  |  |  |
| **Attitude-2** Providing this pharmaceutical care service* allows more patients to come to the pharmacies |  | 0.425 |  |  |  |
| **Attitude-3** Providing this pharmaceutical care service* enhances my professional satisfaction |  | 0.773 |  |  |  |
| **Attitude-4** Providing this pharmaceutical care service* provides rational drug use in older adults |  | 0.726 |  |  |  |
| **Attitude-5** Providing this pharmaceutical care service* provides significant benefits to the health outcomes of older adults |  | 0.791 |  |  |  |
| **Attitude-6** Providing this pharmaceutical care service* enhances older adults’ quality of life |  | 0.766 |  |  |  |
| **Attitude-7** Providing this pharmaceutical care service* enhances the elderly patient’s confidence in pharmacist |  | 0.631 |  |  |  |
| **Attitude-8** Providing this pharmaceutical care service* enhances the physician’s confidence in pharmacist |  | 0.409 |  |  |  |
| **Subjective Norm-1** Physicians think that I should provide this pharmaceutical care service* |  |  | 0.684 |  |  |
| **Subjective Norm-2** Other pharmacists think that I should provide this pharmaceutical care service* |  |  | 0.780 |  |  |
| **Subjective Norm-3** Older adults think that I should provide this pharmaceutical care service* |  |  | 0.564 |  |  |
| **Self-efficacy-1** I have necessary knowledge and skills to provide this pharmaceutical care* |  |  |  | 0.714 |  |
| **Self-efficacy-2** I have necessary [theoretical and practical (case-based)] education to provide this pharmaceutical care* |  |  |  | 0.845 |  |
| **Self-efficacy-3** I have necessary equipment support (like software) to provide this pharmaceutical care service* |  |  |  | 0.591 |  |
| **Perceived Behaviour Control-1***** It is difficult for me to provide older adults with this pharmaceutical care service* |  |  |  |  | 0.619 |
| **Perceived Behaviour Control-2***** It is difficult for me to ask questions to older adults about their medicines and medical conditions during screening for the risk of falls |  |  |  |  | 0.754 |
| **Perceived Behaviour Control-3***** It is difficult for me to identify drug-related problems associated with the risk of falls by conducting medication review (e.g., identifying medication use that increases the risk of falls) |  |  |  |  | 0.691 |
| **Perceived Behaviour Control-4***** It is difficult for me to provide education and counseling service to older adults to reduce the risk of falls |  |  |  |  | 0.769 |
| **Perceived Behaviour Control-5***** It is difficult for me to communicate with the patient’s physicians about the drug-related problem I specify related to the risk of falls |  |  |  |  | 0.677 |
| **Perceived Behaviour Control-6***** It is difficult for me to refer the patient to his/her physician for drug-related problem I specify related to the risk of falls |  |  |  |  | 0.641 |
| **Perceived Behaviour Control-7***** It is difficult for me to follow patients over the period of this pharmaceutical care service* |  |  |  |  | 0.737 |
| **Perceived Behaviour Control-8***** It is difficult for me to electronically record the service I provide within the scope of this pharmaceutical care service* |  |  |  |  | 0.664 |
| **Cronbach alpha** | 0.820 | 0.877 | 0.784 | 0.796 | 0.884 |
| **% of variance** | 8.5 | 15.7 | 7.2 | 7.3 | 15.5 |
| **Median [IQR]** | 4.00 [3.50-4.00] | 4.00 [3.66-4.25] | 3.33 [3.00-4.00] | 3.67 [3.00-4.00] | 3.25 [2.50-3.88] |

* The information about this pharmaceutical care included the definitions and steps of this collaborative deprescribing service for fall-risk medications were presented at the beginning of the survey. **% of total variance= 54.2; KMO: 0.877 (Bartlett’s test of sphericity *p*<0.001); ***reversed items.

**Supplement File Table 3.** ICC coefficients of the domains in the questionnaire according to test-retest reliability (n=30)

| **TPB-based questionnaire** | **ICC (95% CI)** |
| --- | --- |
| **Factor-1 Intention** | 0.914 (0.821-0.959)^a,b^ |
| **Factor-2 Attitude** | 0.891 (0.772-0.948)^a,b^ |
| **Factor-3 Subjective Norm** | 0.841 (0.666-0.924)^a,b^ |
| **Factor-4 Self-efficacy** | 0.905 (0.801-0.955) ^a,b^ |
| **Factor-5 Perceived Behaviour Control** | 0.895 (0.782-0.950) ^a,b^ |

^a^ *p*<0.001; ^b^ Wilcoxon signed rank test used *p*>0.05; ICC: Intra-class Correlation.
